# Impacts of vitamin C and D supplement on COVID-19 treatment: possible patho-mechanisms and evidence from different countries

**DOI:** 10.1186/s43168-023-00186-6

**Published:** 2023-02-20

**Authors:** Sohel Ahmed, Mehrin Hossain, Deepita Chakrabortty, Kazi Ifthi Arafat, Mohammad Jakir Hosen, Mohammad Mizanur Rahman Khan

**Affiliations:** 1 Department of Chemistry, Shahjalal University of Science and Technology, Sylhet, 3114 Bangladesh; 2 Biotechnology Program, Department of Mathematics and Natural Sciences, School of Data and Sciences, BRAC University, Dhaka, Bangladesh; 3 Department of Biotechnology and Genetic Engineering, Bangabandhu Sheikh Mujibur Rahman Science and Technology University, Gopalganj, Bangladesh; 4 Department of Genetic Engineering and Biotechnology, Shahjalal University of Science and Technology, Sylhet, 3114 Bangladesh

**Keywords:** Vitamin C, Vitamin D, COVID-19, SARS-CoV-2, Treatment, Mechanism

## Abstract

**Background:**

A balanced diet and nutrition greatly influence our immune system’s ability and regulate the risk and severity of infections. This review presented the possible patho-mechanisms of vitamins C and D in COVID-19 immunity.

**Main body:**

Deregulation of the immune system including the decreased level of immune boosters is invariably reported in COVID-19. Vitamin C and vitamin D are among the immune boosters; homeostasis of those was found essential for fighting against the viruses, and COVID-19 is no exception. Statistical data strengthens the statements put forth on the effects of these vitamins regarding the complications, symptoms, and mortality.

**Short conclusion:**

A comprehensive literature review revealed that vitamin C helps to reduce and in some cases eradicate the particular symptoms that pose major risks of COVID-19 while balanced vitamin D content in COVID-19 patients has been proved to possess a negative correlation with mortality.

## Background

The COVID-19 pandemic caused by SARS-CoV-2 has rampant the homeostasis of the global economy, politics, and livelihood. More than 100 million people have been affected by this infectious disease with almost 3 million deaths to date [1–4]. The clinical features of COVID-19 are varied from non-symptomatic or moderate illness to severe respiratory problems and possibly death [5]. Deregulation of the immune system including cytokine storms is found to be an important indicator of COVID-19 severity. Immune-boosting therapeutics and diet are found to play an important role to fight COVID-19. The deficiency of immune-boosting vitamins C and D is reported to increase the risk and severity of COVID-19 [6]. Vitamin C and vitamin D supplement stimulates antiviral activity and prevents cytokine storms in COVID-19 patients and results in a good outcome [7].

In this review, we will highlight the underlying mechanisms of vitamin C and vitamin D in boosting antiviral activity and preventing cytokine storms in COVID-19 patients. We also summarize aggregative evidence of both vitamins’ work against COVID-19.

## Main text

### Mechanisms of vitamin C in antiviral activity and preventing cytokine storm

Vitamin C, also known as ascorbic acid, is an essential component for the growth, development, and repair of the body [8]. It acts as a multi-regulator in our body to protect and reduce the severity of various diseases. The immunomodulatory activity of vitamin C plays a role in both innate and adaptive immunity which can protect the physical tissue barrier [9]. It performs an important part in tissue repair by down-regulating the cytokines and safeguarding the endothelium from oxidant injury [9].

**Fig. 1:**
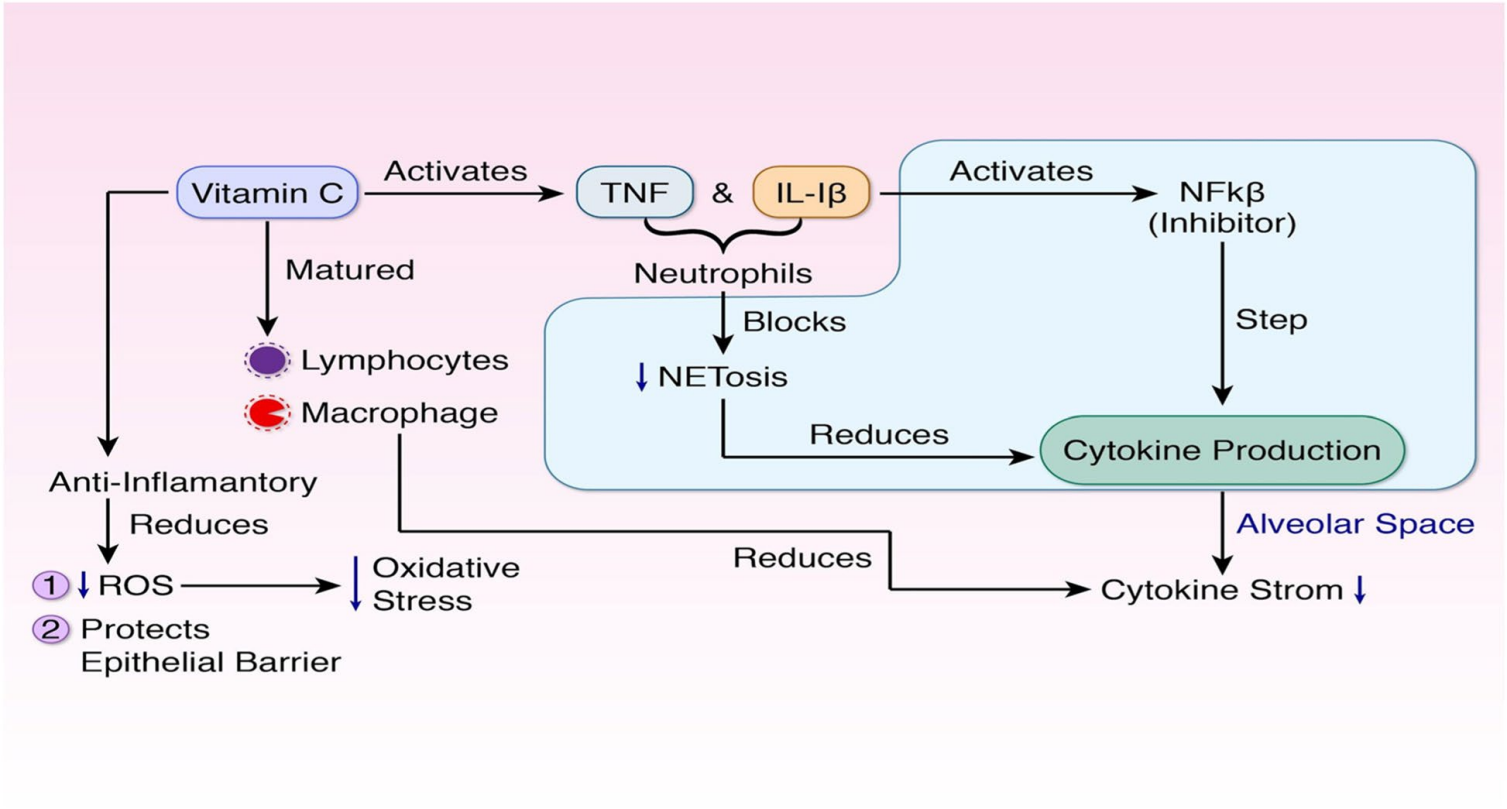
Vitamin C. Its immunomodulatory activities prevent the formation of cytokine storms in two ways. **a** It activates neutrophils (TNF and IL-1β) which block neutrophil extracellular trap (NETosis) in the alveolar space. On the other hand, the neutrophils also inhibit nuclear transcription factor kappa B (NFkB). These two ways prevent the formation of cytokines in the body. **b** Vitamin C also matures lymphocytes and macrophages in the prevention of cytokine storms. Moreover, vitamin C has anti-inflammatory activities, reduces reactive oxidative species (ROS), and protects the epithelial barrier. These activities of vitamin C protect the body from diseases and also reduce the severity of diseases

Vitamin C activates neutrophils including TNF and IL-1β, which activates the inhibitor nuclear transcription factor kappa B (NFkB), and stops the cytokine production in the alveolar space (Fig. 1). On the other hand, activation of TNF and IL-1β blocks NETosis (neutrophil extracellular trap) that hampers the cytokine production in the alveolar space [10]. As a result, a cytokine storm does not take place. Moreover, vitamin C helps in the development and maturation of T lymphocytes during the period of infection [9]. Matured lymphocytes and macrophages also help to decrease the number of cytokines and protect from cytokine storms (10, Fig. 1).

The antioxidant activity of vitamin C reduces oxidative stress by decreasing reactive oxidative species (ROS) [9, 11]. Moreover, the antioxidant activity of vitamin C helps to protect the epithelial barrier [11]. On the other hand, vitamin C acts as a cofactor for many enzymes and helps the biosynthesis of hormones such as norepinephrine, catecholamines, and vasopressin; methylation of DNA; and histones [11]. Vitamin C also has antimicrobial properties (such as antifungal, antibacterial, and antiviral) which protect the body from diseases [12].

## Mechanisms of vitamin D in antiviral activity and preventing cytokine storm

Vitamin D is a fat-soluble vitamin, responsible for calcium homeostasis and bone metabolism. In addition, vitamin D plays an important role in boosting immunity and reducing the severity of many infectious diseases. Vitamin D helps the maturation and differentiation of monocytes and macrophages and plays a role in the production of T lymphocytes [13]. Unfortunately, billions of people around the world are reported to have a deficiency in vitamin D and are prone to many infectious diseases [14].

**Fig. 2:**
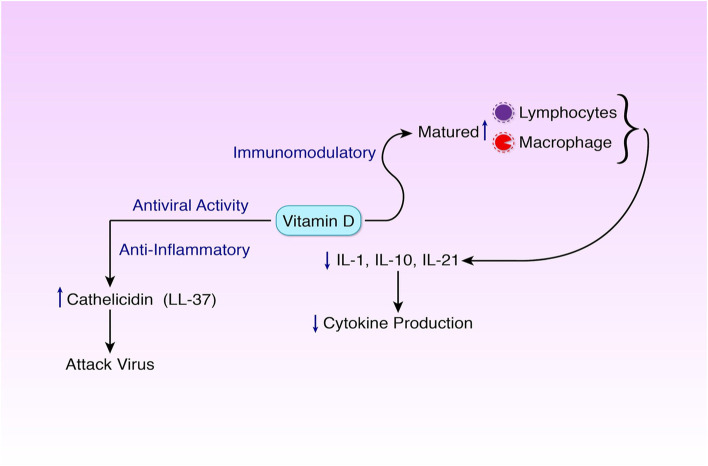
Vitamin D. It has three activities. **a** It has immunomodulatory activity. Here, it matures lymphocytes and macrophage. **b** Its anti-inflammatory activity decreases the level of IL-1, IL-10, and IL-21. As a result, the level of cytokine decreases. **c** In its antiviral activity, vitamin C increases the level of cathelicidin (LL-37) which attacks viruses. Vitamin D helps to protect the body from diseases and reduce the severity of diseases

Vitamin D also has anti-inflammatory features and can inhibit the expression of pro-inflammatory cytokines including IL-10, IL-21, and IL-1 s with help of macrophage and T-cells, as a result, preventing cytokine storms [11, 15] (Fig. 2).

Vitamin D exhibits antiviral activity. When the viral infection increases, vitamin D gets activated and helps increase the production of antimicrobial peptide cathelicidin (LL-37) and reduce the viral infection [13, 16].

## Evidence of vitamin C in reducing COVID-19 severity

The immune-modulatory and antimicrobial activity made vitamin C an important factor in many chronic and infectious diseases [17]. Most studies have reported that a high dose of vitamin C supplement correlated with reduced infection and inflammation susceptibility and lower hospitalization and requirement for mechanical ventilation, as well as reduced mortality (Table 1). On the other hand, a low dose of vitamin C has been shown to cause an insignificantly lower level of thrombosis (Table 1).

**Table 1:** Effectiveness of vitamin C supplement in COVID-19 treatment in different populations

Treatment	Key findings	References
High dose of vitamin C	Reduced inflammation and severity of the disease and improved oxygen saturation	[18, 19]
	No effect on the number of needed ventilation days	[20]
	Increase in oxygenation and reduction in the amount of inflammatory markers were observed	[21–23]
	Reduced overall mortality	[24]
	Lower rates of mechanical ventilation and cardiac arrest	[25]
	Lower rate of hospitalization	[26]
	No significant outcomes	[27–29]
Low dose of vitamin C	No difference was found in the case of mortality rate and the need for mechanical ventilation	[30], [5, 31–33]
	Lower level of anti-inflammatory response observed	[34, 35]
	Lower rate of thrombosis	[30]

## Role of vitamin D

The optimum performance of our immune system is incredibly influenced by vitamin D. Its insufficiency has been associated with a vulnerability to respiratory infections [36, 37]. The necessary amount of vitamin D for our bodies is obtained via food supplements or sunlight. 7-Dehydrocholesterol, the precursor to vitamin D, becomes active when sunlight contacts the skin, and the ultraviolet B (UVB) rays then transform it into vitamin D3. Vitamins D2 and D3 are transformed by our liver into 25-hydroxyvitamin D3 [25(OH)D3]. COVID-19 patients with vitamin D deficiency exhibited increased infection and susceptibility, higher mortality, and increased ARDS. Vitamin D supplement has been found to lower viral multiplication and lower susceptibility and inflammation and ICU need (Table 2).

**Table 2:** Effectiveness of vitamin D supplement in COVID-19 treatment in different populations

Treatment	Key findings	References
Vitamin D deficiency	Increased mortality	[36, 38]
	Increased COVID-19 susceptibility	[39]
	Intensified acute respiratory distress syndrome (ARDS)	[39]
	Increased rate of infection	[40]
Vitamin D supplement	Lower rate of ICU need	[41]
	Lower level of fibrinogen	[42]
	Lower level of ARDS	[39]
	Lower severity	[43]
	Increased level of anti-inflammatory cytokines and decreases levels of pro-inflammatory cytokines, boosts the production of natural antimicrobial peptides, and activates cells like macrophages that can kill SARS-CoV-2	[39]
	Increased human cationic antimicrobial protein (hCAP18) in pregnant women, play role in the post-SARS-CoV-2 infection treatment	[17]
	Positively impacts on the innate and adaptive immune system. Significantly reduce pro-inflammation by selectively inhibiting inflammatory cytokines, decreasing leukocyte infiltration, increasing memory and regulatory T cells, and lowering the lymphocyte ratio	[44]
	Lower viral multiplication	[45]

## Summary

Compilation of the comprehensive literature studies revealed that supplementation of vitamin C and vitamin D at an early stage of COVID-19 infection is highly beneficial for patients. Interestingly, both the vitamin C and vitamin D supplement work better if given in high doses for a long period. More critical and hospitalized COVID-19 patients were also found associated with vitamin C and vitamin D deficiency. However, studies undertaken by different groups on various populations report heterogenous outcomes of vitamin C and D supplements. Additionally, different physicians prescribed different doses and courses of vitamins C and D, which indicates the requirement for precise guidelines. The studied population size was also too small to make any concrete decision. Intravenous supplementation of vitamin C and vitamin D works better than taken orally. Efficacy and safety are the two major concerns for treating COVID-19 patients with vitamins C and D. As the rate of vitamin C metabolism in blood is high, larger doses along with longer courses of vitamin C are recommended. A high dose of vitamin C has been used for decades. Recently, an NIH expert panel states that a high dose of vitamin C (1.5 g/kg body weight) is safe without major adverse effects (https://www.cancer.gov/about-cancer/treatment/cam/hp/vitamin-c-pdq). None of the trials revealed any major side effects of using high doses of vitamins C and D in the patient’s body. On the other hand, it is obvious that the result may vary with different populations and patients with other comorbidities [46–49]; thus, more detailed studies are required. Apart from the COVID-19 treatment, vitamin C is generally safe. But for some people, it may cause stomach cramps, nausea, and headache. Taking more than 2000 mg/per day is unsafe and may cause kidney stones and severe diarrhea. Similarly, a high dose of vitamin D can cause stomach discomfort, unusual symptoms, or kidney problems. Thus, COVID-19 patients are prescribed to take vitamins C and D as per doctor’s advice.

## Conclusions

Mode of administration, dosage, initiation time, treatment duration, disease type, and disease progression play an effective role in the results of the studies regarding the effectiveness of vitamin C and vitamin D on COVID-19. Despite not getting any significant results, there is still a possibility to find the effectiveness of these two vitamins on COVID-19 as the results were not fully negative. As no significant side effects were observed, vitamin C and D supplement may be advised specially for hospitalized critical COVID-19 patients.

## Data Availability

Not applicable.
